# Adeno-associated virus-based gene therapy for hemophilia–addressing the gaps

**DOI:** 10.1016/j.rpth.2024.102673

**Published:** 2024-12-31

**Authors:** Wolfgang Miesbach, Paul Batty, Pratima Chowdary, Sylvia Fong, Radoslaw Kaczmarek, Frank W.G. Leebeek, Brian Long, Johnny Mahlangu, Mike Makris, Glenn F. Pierce, Steven W. Pipe, Alok Srivastava, Jan Voorberg, Flora Peyvandi

**Affiliations:** 1 Medical Clinic 2, University Hospital Frankfurt, Frankfurt, Germany; 2 Department of Haematology, Cancer Institute, University College London, London, United Kingdom; 3 Katharine Dormandy Haemophilia and Thrombosis Centre, Royal Free Hospital, London, United Kingdom; 4 Queen’s University, Kingston, Ontario, Canada; 5 Herman B Wells Center for Pediatric Research, Indiana University School of Medicine, Indianapolis, Indiana, USA; 6 Ludwik Hirszfeld Polish Academy of Sciences, Institute of Immunology and Experimental Therapy, Wroclaw, Poland; 7 Department of Haematology, Erasmus University Medical Center, Rotterdam, the Netherlands; 8 4D Molecular Therapeutics, Emeryville, California, USA; 9 Department of Molecular Medicine and Haematology, Faculty of Health Sciences, University of the Witwatersrand, Johannesburg, South Africa; 10 School of Medicine and Population Health, University of Sheffield, Sheffield, United Kingdom; 11 Worf Federation of Hemophilia, Montreal, Québec, Canada; 12 University of Michigan, Ann Arbor, Michigan, USA; 13 St. John’s Research Institute, St. John’s Medical College Hospital, Bengaluru, India; 14 Sanquin Research, Amsterdam, the Netherlands; 15 Fondazione IRCCS Ca’ Granda Ospedale Maggiore Policlinico, Angelo Bianchi Bonomi Hemophilia and Thrombosis Center, Milan, Italy; 16 Department of Pathophysiology and Transplantation, Università degli Studi di Milano, Milan, Italy

**Keywords:** gene therapy, hemophilia, methods, safety, standardization

## Abstract

Adeno-associated virus-based gene therapy for hemophilia has emerged as a revolutionary treatment option, offering potential correction of clotting factor deficiency through a single intravenous infusion of functional genes directed to hepatocytes. With 3 gene therapies recently approved, this approach shows promise in transforming the lives of individuals with hemophilia. However, the complexity of gene therapy and the lack of standardization of methods in different components of this therapy can lead to unique challenges for clinical implementation. This manuscript follows literature reviews and structured discussions by the International Society on Thrombosis and Haemostasis Scientific and Standardization Committee Working Group on Gene Therapy that identified specific areas requiring standardization of methods, including viral vector production, liver function assessment, quantification of factor (F)VIII and FIX expression levels, assessment of antiadeno-associated viral antibodies, and genomic integration detection methods. Standardization strategies aim to achieve consistent vector quality, effective patient selection, and uniform assessment methods by implementing advanced laboratory techniques and standardized protocols. Standardizing these parameters is essential for improving the understanding of short-term and long-term safety and efficacy of gene therapy in hemophilia. This effort aims to enhance the predictability of individual responses, address variability in outcomes, and ultimately provide more effective, safer, and personalized treatment options for individuals with hemophilia.

## Essentials

• Complexity of gene therapy and the lack of standardized methods present unique challenges.
• After discussion at the Gene Therapy Working Group of the ISTH, there are crucial areas requiring standardization.
• Standardizing is essential for improving understanding of the variability of response, as well as short- and long-term safety.

## 1 Introduction

Delivering functional F8 and F9 genes to hepatocytes via adeno-associated viral (AAV) vectors through a single intravenous infusion marks a significant advancement in the treatment of hemophilia A and B [1]. More than 400 individuals with hemophilia have participated in clinical gene therapy studies so far, including more than 300 participants across 4 phase 3 trials (the European Association for Haemophilia and Allied Disorders gene therapy database) [2]. This approach has resulted in sustained production of transgene-derived clotting factors and a marked reduction in bleeding events. In the first intravenously administered AAV8-based gene therapy study for hemophilia B, factor (F)IX levels remained stable over a median follow-up of 10.7 years (range, 4-12 years). In the highest dose cohort (2 × 10^12^ vg/kg), 6 participants had a mean (±SD) FIX activity of 4.9 ± 2.2 IU/dL (one-stage [OS] assay). This corresponded to a 21-fold decrease in the annual bleeding rate (mean annual bleeding rate, 1.16; median, 1), which was statistically significant (paired *t*-test, *P* < .008) [3].

The first clinical trial using the high-active FIX Padua variant was published in 2017 [4].

For gene therapy of hemophilia A, at years 7 and 6, mean (median) FVIII activity (chromogenic substrate [CS] assay) was 16.2 (10.3) and 6.7 (7.2) IU/dL in the 6 × 10^13^ (*n* = 5) and 4 × 10^13^ (*n* = 4) cohorts, respectively, corresponding to mild hemophilia and leading to a 96% and 88% decrease of the annual bleeding rate from baseline, respectively [5].

Prior to initiating gene therapy, a comprehensive evaluation of liver health is essential, including the exclusion of active hepatitis [6,7], uncontrolled HIV infection, and inhibitors to coagulation factors, as well as testing for anti-AAV antibodies [8]. Patient eligibility for currently licensed AAV vector therapies is product-specific: valoctocogene roxaparvovec (Roctavian, BioMarin Pharmaceutical) [9] is indicated for adult males with severe hemophilia A, requiring the absence of antibodies against AAV5 as well as fidanacogene elaparvovec for adult males with moderate to severe hemophilia B (Beqvez, Pfizer Canada ULC) [10].

Etranacogene dezaparvovec (Hemgenix, CSL Behring) [11] is suitable for adults with severe and moderate hemophilia B. Although the presence of antibodies to AAV does not universally preclude patients from treatment with etranacogene dezaparvovec, it may, dependent on their titer, significantly impact the vector’s ability to reach and transduce hepatocytes when delivered systemically.

Gene therapies for hemophilia exhibit significant variation in their AAV capsid serotypes, expression cassettes, and vector dosages. These dosages can differ by a factor of 300, ranging from 2 × 10^11^ vector genomes per kilogram (vg/kg) to 6 × 10^13^ vg/kg [12].

Studies have shown significant interindividual variability in factor expression levels, making it difficult to precisely predict factor expression and long-term response [12]. The transduction pathway of hepatocytes involves multiple complex steps, each with variable efficiency among patients, which may contribute to the observed variability in therapeutic outcomes [1].

After approval, the implementation of gene therapy is impeded by the intricate nature of the therapy and the absence of standardized protocols for several critical gene therapy-related parameters and assessments. This communication follows discussions by the International Society on Thrombosis and Haemostasis (ISTH) Scientific and Standardization Committee (SSC) Working Group on Gene Therapy and has been endorsed by the ISTH SSC, aiming to define these challenges and provide guidance on essential parameters requiring standardization. This effort will facilitate consistent and predictable therapeutic outcomes and improve the monitoring of adverse events.

## 2 Strategies for Standardization of Methods

Following the approval of the first 3 gene therapies for hemophilia, a comprehensive approach to standardizing methods is critical. This will address the challenges associated with gene therapy identified in clinical trials to date. Importantly, by developing standardized protocols, comparability between different studies and clinical trials can be achieved, which is essential for the collection of robust and generalizable data. This uniformity of procedures and evaluations helps to minimize variables that can lead to inconsistent results and safety issues, ultimately advancing gene therapy techniques and improving the quality of life for individuals with hemophilia. Standardization should encompass methods for manufacturing, patient selection, and follow-up processes.

Table 1 summarizes the key challenges in gene therapy for hemophilia and proposes strategies to address these complexities, aiming to optimize treatment outcomes and ensure patient safety.

**Table 1:** Challenges in gene therapy for hemophilia.

Challenges in gene therapy for hemophilia	Strategies to overcome challenges
Influence of anti-AAV antibodies	- Conduct comprehensive pretreatment assessments for anti-AAV antibodies to determine patient suitability - Develop more refined and standardized assays for antibody detection and classification - Develop guidance on how certain levels can be interpreted
Importance of liver health	- Ensure thorough liver function evaluation, both pre- and posttreatment - Monitor and manage any side effects effectively to maintain liver health
Short-term safety concerns	- Develop guidance on the management of infusion-related reactions - Identification of ALT elevation significant to transgene expression - Initiate immediate immunosuppressive therapy in cases of early liver enzyme elevation
Durability of response	- Conduct long-term follow-up studies to monitor factor levels and therapeutic effectiveness - Explore and develop alternative gene therapy approaches that may offer longer-lasting effects
Long-term safety concerns	- Establish and maintain comprehensive registries for tracking long-term safety data - Conduct in-depth studies to understand the risks of gene integration and potential tumorigenesis - Develop and implement rigorous posttreatment surveillance protocols

AAV, adeno-associated virus; ALT, alanine transaminase.

### 2.1 Viral vector products

The production of viral vectors utilizes diverse cell types, each offering unique advantages and posing specific challenges. Three cell lines, including 2 human (human embryonic kidney [HEK] and HeLa) and one insect (Sf9), have been used [13]. Mammalian cells, such as HEK cells (HEK293), are human cells, potentially yielding vectors that are more compatible with human physiology. However, insect cell lines present benefits in scalability and a lower risk of contamination with human pathogens. Sf9 cells have their own contamination issues [14]. These differing cell types underscore the need for a nuanced approach to vector production [15].

Standardization of the production process using different cell lines is critical to ensure consistent quality and efficacy of the vectors [16]. Standardization should focus on several key aspects: first, the process must address the variability in vector production. Factors such as purity, contaminants, and the ratio of full to empty viral particles can vary between batches, directly impacting the safety and efficacy of the therapy. Therefore, a robust monitoring and control system is essential to maintain the integrity of the production process. Second, the scalability of production poses a significant challenge.

Advanced production techniques are imperative to tackle these challenges. The implementation of state-of-the-art biotechnological methods and automation can enhance the consistency and efficiency of vector production. This includes improving cell line engineering for more stable and efficient vector production, optimizing vector purification processes to increase yield and purity, and employing rigorous quality control measures throughout the production process [13].

Furthermore, stringent quality control measures are essential. Establishing strict protocols for regular monitoring of vector potency, purity, and safety ensures that each production batch meets the required standards, thus guaranteeing the reliability and effectiveness of the therapy.

### 2.2 Assessing anti-AAV antibodies

AAV antibodies present a considerable obstacle in the field of gene therapy. A significant proportion of the population, including many candidates for gene therapy, possess neutralizing antibodies targeting the AAV capsid. This phenomenon is attributed to previous (asymptomatic) infections with wild-type AAV, with prevalence varying according to geographic location and age [17]. These naturally occurring antibodies hinder the success of gene therapy by obstructing the AAV vectors’ ability to deliver therapeutic genes to target cells.

The impact of these antibodies is particularly critical when the vector is administered systemically in the circulation. However, empirical evidence suggests that direct injections into solid tissues or the subretinal space are not impeded by the presence of these antibodies [18], and administering very high vector doses may overcome the effects of these antibodies, with progressively higher doses of empty particles shown to mitigate the impact of neutralizing antibodies [19].

Assessing immunogenicity is a complex process involving various methodologies. Several tests are performed to determine the total antibody level or neutralizing antibodies. These include enzyme-linked immunosorbent assays, radioimmunoassays, and cellular assays like transduction inhibition assays, ELISpot, or flow cytometry [20]. These assays detect antibodies and cellular immune responses against the viral vector, and also potential responses to the transgene-expressed protein. Functional inhibitor assays (Bethesda or the Nijmegen-Bethesda assay) are necessary to monitor for antibodies to the transgene-expressed FVIII or FIX.

Each method has its unique approach and utility. Enzyme-linked immunosorbent assays are widely used for their sensitivity and specificity in measuring total antibody levels. Although less common, radioimmunoassays provide high sensitivity and are useful in quantifying specific antibodies. Cellular assays like ELISpot and flow cytometry are crucial for evaluating T-cell responses, providing insights into the cellular immune response against the gene therapy vector or the transgene.

Though several manuscripts and guidance documents have been produced on the standardization of methods and analysis for immunoassays [[21], [22], [23]], the format and utilization of these assays have been applied unevenly, making comparisons across studies challenging. To address this, there is a need to harmonize methods for evaluating immunogenicity. This should involve defining clear cutoff values for what constitutes a positive immune response, ensuring the timing of sample collection is consistent across studies, and employing uniform assay protocols. For cellular assays, peripheral blood mononuclear cell collection, cryopreservation, storage, and shipment should also be standardized.

Establishing international standards for the detection of anti-AAV antibodies would include assay sensitivity, specificity, and diagnostic accuracy assessment. This would enable increased interchangeability between assays as well as comparability between the different therapies.

As an example of variation due to different assay parameters, transduction inhibition assays performed in early studies of etranocogene dexaparvovec and valoctocogene roxaparvovec used widely discrepant multiplicities of infection (the number of viral particles per cell), which would yield more than 60-fold different titers for the same vector [24].

Contrary to all other gene therapies for hemophilia, the anti-AAV antibody status has to be checked before the treatment with etranocogene dexaparvovec but is not an exclusion criterion for treatment [25]. The clinical relevance of titers below a certain threshold is still unclear. According to the Food and Drug Administration Summary Basis for Regulatory Action [11], the mean ± SD FIX activity at month 12 was 42% ± 22% in subjects with titers ≤1:350 (*n* = 47) and 27% ± 17% in subjects with titers >1:350 to <1:700 (*n* = 3). However, the anti-AAV antibody titer assay used in these studies has not been validated, and therefore, the results should be interpreted with caution. Consequently, the Food and Drug Administration is required to develop and validate a sensitive and accurate assay for the detection of preexisting anti-AAV5 neutralizing antibodies and a postmarketing study evaluating at least 35 persons with hemophilia B screened with the validated assay [11].

As a first step, the ISTH SSC Working Group on Gene Therapy collected information from the pharmaceutical industry about the type of anti-AAV tests. The collection of efficacy data over the long term will also be useful in order to correlate levels of transgene expression with levels of preexisting antibodies.

Guidelines for interpreting these results are needed to facilitate patient eligibility selection and enhance comparability across studies and trials. This will also contribute to an improved understanding of the interactions between AAV vectors and the human immune system.

### 2.3 Liver dysfunction and interventions post gene therapy

Liver health should be assessed during the initial screening to determine eligibility and evaluate the risk of hepatocellular carcinoma. After gene therapy, ongoing monitoring for liver inflammation, overall liver health, and screening for hepatocellular carcinoma are essential to verify long-term safety.

Evaluation of liver health currently uses laboratory measurements of liver enzymes and noninvasive imaging assessments for fibrosis, such as Fibroscan and ultrasound.

Currently, there is no data available to provide guidance on whether these assessments should be the same for those with a previous history of HIV and hepatitis and those who have never experienced viral infections. In addition, how changes related to antecedent viral infection or nonalcoholic fatty liver disease can be distinguished from changes related to vector administration. Close cooperation between hemophilia treaters and hepatologists has been proposed [6,7]. Existing approaches to evaluate liver function face challenges in consistency due to variability in the timing of tests, methodology, reference ranges, and the interpretation of results across different clinical settings. There is a need to standardize approaches to evaluate liver function before and after gene therapy. This standardization should establish specific time points for testing. For instance, blood testing (ALT and aspartase transferase) should be conducted at regular intervals–such as pretreatment, immediately posttreatment, and at subsequent follow-up visits (eg, weekly after gene therapy for the first 3 months, at 6 months, and annually posttreatment). This would provide a comprehensive understanding of the liver’s response to the therapy over time.

Differences in protocols on how to use immunosuppressive agents (prophylactically or reactive in case of increased ALT) may not always faithfully represent differences between gene therapies (eg, vector immunogenicity, quality, etc.) because, in the different studies, different thresholds for initiating immunosuppression have been used. To date, there is no evidence that the prophylactic use of corticosteroids or other immunosuppressive agents can reliably prevent an increase in ALT [26].

Defining standardized threshold values for test results is crucial. For ALT and aspartase transferase, establishing what constitutes normal, borderline, and high levels will enable clinicians to interpret results consistently. Hereby, the intraindividual variation of ALT should be considered. Uniform interpretation is vital for assessing the extent of liver function, impairment, or recovery following gene therapy. However, the wide variation of the clearance of circulating ALT [27] makes the definition of a threshold value for initiating treatment challenging, particularly in the early weeks after vector administration, when changes in ALT are likely to be dynamic and transient.

Implementing noninvasive imaging tests like transient elastography (eg, Fibroscan) should be standardized in terms of both execution and interpretation. Fibroscan is instrumental in assessing liver stiffness as an indicator of fibrosis or cirrhosis, which can impact the liver’s ability to process and respond to gene therapy. One of the advantages of the Fibroscan is that there is less variability between operators than with abdominal ultrasound. Standardized protocols for conducting Fibroscan at certain time points and interpreting its results are needed to ensure consistency in evaluating liver health across various clinical environments.

### 2.4 Evaluation of factor levels

Given the complexity of the transduction process of the hepatocytes and the many steps that can potentially vary individually from patient to patient, there is a high degree of variation in the transgene expression. For example, in participants of the HOPE-B study with undetectable baseline neutralizing antibodies to AAV5, the FIX levels at 18 months ranged from 5% to 112%, resulting in a 22-fold range [25]. Similar variability was seen in gene therapy trials in persons with hemophilia A. A reduction in transgene expression over time was observed in participants in the hemophilia A gene therapy trials [28].

The accurate measurement of FVIII and FIX activity is critical in evaluating gene therapy’s efficacy. Discrepancies between OS and CS assays present a significant challenge in standardizing these measurements. In hemophilia A, for instance, transgene-produced FVIII (FVIII-SQ) exhibits higher activity in OS assays compared with CS assays [29]. This difference is notable because recombinant FVIII-SQ products demonstrate an opposite discrepancy (lower OS than CS activity). There is also an impact of different coagulation factor assays using the FIX Padua variant.

This variation in assay results has important implications for patient care. In a clinical setting, measuring both endogenous (naturally occurring) and exogenous (infused or gene therapy-derived) factors is essential. The OS assay, while widely used, may not always provide the specificity needed, particularly in the context of gene therapy where modified forms of clotting factors are introduced. The CS assay offers a more specific quantification but may not be as readily available or standardized across different laboratories. Given these complexities, there is a pressing need for standardization in assay methods to ensure consistency and reliability in measuring factor levels. This would involve selecting the most appropriate assay type with standardized procedures, reagents, and equipment. Such standardization and the use of external quality control (such as ECAT) would enable reliable comparisons of efficacy data across different studies and treatment centers, providing a clearer understanding of the effectiveness of gene therapy and finally allowing the development of guidance for treatment.

At this point, a recommendation around which factor assay(s) to use would seem to be premature. Large datasets are needed to study the association between bleeding frequency and factor levels. These should be determined by different assays for these novel proteins, either FIX Padua or B domain-deleted FVIII-SQ made in human hepatocytes since they have not been used as coagulation factor concentrates.

The preexisting condition of the patient’s joints might be another key indicator of overall joint bleeding events posttreatment, along with other relevant clinical endpoints, such as quality of life.

### 2.5 Methods to investigate the AAV integration frequency

There is a theoretical risk of insertional mutagenesis from AAV that could act as one step in the process of tumorigenesis that has not been reported to date in large animal models or clinical studies. Recently, the vector persistence has been described in a dog model more than 1 decade after gene therapy [30], with data from preclinical and clinical studies recently reviewed by Batty et al. [31]. The predominant vector form was nonintegrated episomal structures with levels correlating with long-term transgene expression. No full-length integrated vectors were found, but random integration was seen in all samples with small numbers of nonrandom common integration sites associated with open chromatin [30]. Despite integration, this was not associated with oncogene upregulation or histopathological evidence of tumorigenesis.

Potential mechanisms via which integration events could provide one step in the process of tumorigenesis are based on reports from retroviral/lentivirus studies. These include increased gene expression by the promotor/enhancer activity or via 3′ end truncation [32]. Another mechanism of tumorigenesis could be interruption by integrating vectors of the expression of a tumor suppressor gene.

The limited number of noncommercial laboratories equipped to conduct these assays underscores the urgency for standardization. Developing high-throughput standardized integration assays will allow comparability across different studies, crucially advancing gene therapy research and improving safety and application. This move toward a more inclusive and standardized approach in gene therapy for hemophilia is a scientific necessity and a step toward greater transparency and collaboration in this field.

Although there have been no cases of tumors related to AAV reported to date, there needs to be robust protocols in place to allow accurate assessment of whether this could be related to AAV. International standards for testing and evaluation include establishing uniform protocols for integration assays to ensure consistent results across different laboratories. It should be acknowledged that differences may exist based on the type of tumor, location, limited sample size, and tissue integrity. It should be defined how samples are stored and which assays should be used. Moreover, creating global reference centers for performing and interpreting these assays is essential, contributing to a broader understanding of gene therapy’s implications. There are a number of advanced assays using either target enrichment sequencing or linear amplification-mediated polymerase chain reaction-based approaches in the research setting with different advantages and limitations. The precision of next-generation sequencing in detecting and characterizing AAV vector integration is crucial.

Furthermore, the review and assessment of gene integration results should follow public and transparent criteria, enabling more laboratories to conduct these investigations. This approach will allow access to critical assays, extending the capacity for gene therapy evaluation and research beyond specialized centers.

The outlined key elements in Table 2 demonstrate the importance of this comprehensive approach.

**Table 2:** Various strategies for standardization in gene therapy of hemophilia.

Gene therapy process	Standardization approach	Future aim
Viral vector production	• Viral vector production utilizes different cell types, each offering unique benefits and obstacles. • The production process needs to effectively manage variations in vector quality and address scalability issues. • Implementation of advanced production methods is crucial, including better cell line engineering and optimized vector purification.	Achieve consistent vector quality and biological comparability.
Assessment of anti-AAV antibodies	• Immunogenicity assessment employs various techniques such as ELISAs, radioimmunoassays, and cellular assays. • These methods differ in their methodologies and applications, highlighting the need for standardization to ensure uniformity in clinical trials. • Establishing clear cutoff values, maintaining consistency in the timing of sample collection, and adopting uniform assay protocols are fundamental requirements.	Effective patient selection processes and a deeper comprehension of AAV vectors’ interactions with the immune system.
Evaluation of liver function	• Liver function assessment in gene therapy includes measuring enzymes like ALT and aspartase transferase and conducting imaging tests such as Fibroscan. • Standardizing the timing of these tests, the methodologies used, and the interpretation of results is essential for consistency. • It is important to establish specific time points for conducting these tests and to define standardized threshold values for interpreting results.	Standardize testing protocols and limit values to consistently evaluate liver health and gauge the impact of gene therapy accurately.
Evaluation of efficacy and factor levels	• Accurate measurement of clotting FVIII and FIX is key in evaluating gene therapy efficacy for hemophilia A and B. • Discrepancies between OS and CS assays necessitate standardization for consistent and reliable factor-level measurement. • This involves selecting appropriate assay types and standardizing procedures, reagents, and equipment, ensuring reliable efficacy data comparison, and understanding gene therapy effectiveness.	Standardize efficacy measurement assays.
Integration methods	• Standardization of integration methods for AAV vectors is vital for safety assessment in gene therapy. • Uniform protocols for integration assays are necessary for consistent results. • Global reference centers for performing and interpreting these assays, along with public and transparent criteria for gene integration review, are essential.	High-throughput, standardized integration assays are crucial for advancing research and ensuring inclusive and transparent approaches in gene therapy for hemophilia.

AAV, adeno-associated virus; ALT, alanine transaminase; AST, aspartase transferase; CS, chromogenic substrate; ELISA, enzyme-linked immunosorbent assay; FIX, factor IX; FVIII, factor VIII; OS, one-stage.

## 3 Conclusion

The ISTH SSC’s Gene Therapy Working Group emphasizes the importance of standardizing methods related to gene therapy for hemophilia. This includes streamlining processes for viral vector production, liver function tests, and the measurement of clotting FVIII and FIX. Additionally, the group focuses on standardizing methods for assessing immune responses and genome integration, which is crucial for tracking potential implications of gene integration and evaluating immunogenicity in clinical trials. Efforts are also being made to establish detailed standardization guidelines and conduct extensive field studies, all aimed at improving the efficacy and safety of gene therapy for hemophilia.

Collaboration across disciplines is crucial. To bridge the gap between laboratory research and clinical application, researchers and clinicians must work together, focusing on molecular aspects and practical challenges of gene therapy for hemophilia. This collaboration fosters the development of gene therapies that are both effective and safe, tailored to the diverse needs of patients. Regulatory bodies play a critical role in ensuring new therapies are not just effective but also safe.

A significant aspect of this standardization is focusing on long-term outcomes and exploring alternative gene therapy or gene editing approaches. Conducting extensive long-term studies to understand gene therapy’s sustained effects and potential late-onset complications is imperative. This includes monitoring for delayed adverse effects, assessing the duration of therapeutic benefits, and exploring the need for repeat dosing. Additionally, investigating alternative gene delivery methods and novel vectors with improved safety profiles can provide additional options for hemophilia treatment.

In conclusion, the future of gene therapy for hemophilia relies on a concerted effort from all stakeholders involved in its development and application. Through collaborative research, standardized international practices, and a deepened understanding of gene therapies’ molecular dynamics, the field can advance toward offering more effective, safer, and personalized treatment options for individuals living with hemophilia.
